# The effects of cocoa on the immune system

**DOI:** 10.3389/fphar.2013.00071

**Published:** 2013-06-04

**Authors:** Francisco J. Pérez-Cano, Malen Massot-Cladera, Àngels Franch, Cristina Castellote, Margarida Castell

**Affiliations:** ^1^Departament de Fisiologia, Facultat de Farmàcia, Universitat de BarcelonaBarcelona, Spain; ^2^Institut de Recerca en Nutrició i Seguretat Alimentària, Universitat de BarcelonaBarcelona, Spain

**Keywords:** cocoa, lymphocyte, macrophage, antibody, cytokine, gut-associated lymphoid tissue, thymus, spleen

## Abstract

Cocoa is a food relatively rich in polyphenols, which makes it a potent antioxidant. Due to its activity as an antioxidant, as well as through other mechanisms, cocoa consumption has been reported to be beneficial for cardiovascular health, brain functions, and cancer prevention. Furthermore, cocoa influences the immune system, in particular the inflammatory innate response and the systemic and intestinal adaptive immune response. Preclinical studies have demonstrated that a cocoa-enriched diet modifies T cell functions that conduce to a modulation of the synthesis of systemic and gut antibodies. In this regard, it seems that a cocoa diet in rats produces changes in the lymphocyte composition of secondary lymphoid tissues and the cytokines secreted by T cells. These results suggest that it is possible that cocoa could inhibit the function of T helper type 2 cells, and in line with this, the preventive effect of cocoa on IgE synthesis in a rat allergy model has been reported, which opens up new perspectives when considering the beneficial effects of cocoa compounds. On the other hand, cocoa intake modifies the functionality of gut-associated lymphoid tissue by means of modulating IgA secretion and intestinal microbiota. The mechanisms involved in these influences are discussed here. Further research may elucidate the cocoa compounds involved in such an effect and also the possible medical approaches to these repercussions.

## INTRODUCTION

In addition to the beneficial effects on oxidative stress, cardiovascular health, nervous system diseases, aging, and cancer prevention, cocoa has been revealed as a food with immunoregulatory properties. In the following sections, the influence of cocoa or its flavonoids on the innate and adaptive immunity are reviewed. Firstly, the anti-inflammatory properties of cocoa are briefly summarized, and secondly, the effects of cocoa on the adaptive immune system and intestinal immunity are reported.

To achieve this objective, a systematic search in SCOPUS-V.4 (Elsevier) – SciVerse was conducted for the following key terms: “cocoa” AND “lymphocyte” OR “immun*” OR “inflammation” OR “microbiota.” In order to prevent potential misclassification of relevant articles no exclusion criteria were used. The search included the period from January 1990 to March 2013.

## ANTI-INFLAMMATORY POTENTIAL OF COCOA

Inflammation is the response of tissues to an aggression caused by pathogens, chemicals or wounding. Inflammation involves a complex network of reactions initially designed to protect the host from injury and to heal damaged tissue. The activation and migration of leukocytes to the site of the lesion and the release of growth factors, cytokines, reactive oxygen species (ROS), and nitric oxide (NO) are known to play a crucial role in the inflammatory response. Constant overproduction of pro-inflammatory molecules leads to chronic inflammation.

In general, flavonoids are associated with anti-inflammatory properties. In this regard, the flavanols contained in cocoa have been the subject of both *in vitro* and *in vivo* studies (reviewed in Pérez-Cano et al., 2010). Many studies have reported cocoa’s ability to reduce cytokines, chemokines, ROS, NO, etc. involved in inflammatory response. However, few studies have focused on the *in vivo* anti-inflammatory activity of cocoa.

### COCOA EFFECTS ON INFLAMMATORY CELLS

Cocoa extracts or single flavonoids, both as monomers (epicatechin, catechin) or polymers (procyanidins) have demonstrated *in vitro* their anti-inflammatory potential, although there have been some controversial results.

A cocoa flavonoid-enriched extract and the monomers epicatechin and isoquercitrin were able to decrease the production of inflammatory molecules such as tumor necrosis factor (TNF)-α and monocyte chemoattractant protein (MCP)-1 by macrophages under stimulation with lipopolysaccharide (LPS; Ramiro et al., 2005a). Similarly, epicatechin in stimulated whole blood cells culture suppressed the production of interleukin (IL)-6 and IL-8 (Al-Hanbali et al., 2009). However, monomer to pentamer units and longer chain fractions of cocoa flavanols increased the secretion of TNF-α, IL-1, and IL-6 in LPS-stimulated peripheral blood mononuclear cells (PBMC; Mao et al., 2002; Kenny et al., 2007; Wisman et al., 2008).

Aside from cytokines, other inflammatory molecules can be influenced by cocoa. Epicatechin, procyanidin B_1_, procyanidin B_2_, and a cocoa extract reduced NO release by stimulated macrophages (Ono et al., 2003; Ramiro et al., 2005a; Hämäläinen et al., 2007). Likewise, the *in vitro* treatment with cocoa fractions or flavonoids alone decreased the production of ROS from several kinds of cells (Sanbongi et al., 1997; Erlejman et al., 2006; Granado-Serrano et al., 2007; Ramiro-Puig et al., 2009).

Neutrophils also play an important role during inflammation. It has been demonstrated that cocoa has the potential to positively modulate the neutrophil inflammatory activity. In this sense, certain flavanols and procyanidins isolated from cocoa moderated some signaling pathways induced by LPS on neutrophils, particularly those of oxidative bursts and activation markers, and cocoa could influence selected apoptosis mechanisms (Kenny et al., 2009).

Regarding the mechanisms of action, it has been reported that hexameric cocoa procyanidins have the capacity to modulate TNF-α-induced NF-κB (nuclear factor kappa-light-chain-enhancer of activated B cells) activation in intestinal epithelial cells (Erlejman et al., 2008). NF-κB is a transcription factor involved in the regulation of genes encoding cytokines (IL-1, IL-2, IL-6, IL-8, TNF-α, among others), adhesion molecules (e.g., intercellular adhesion molecule 1, vascular cell adhesion molecule 1, and endothelial leukocyte adhesion molecule 1), acute phase proteins, inducible enzymes [inducible NO synthase (iNOS) and cyxlooxygenase 2 (COX-2)], etc. (Pahl, 1999).

### ANTI-INFLAMMATORY POTENTIAL OF COCOA IN HEALTHY CONDITIONS

In a more physiological approach, using cells isolated from humans or animals fed with diets containing cocoa, the *in vitro* ability to produce inflammatory mediators and the serum concentrations of inflammatory molecules has been studied.

Some studies have focused on the *in vitro* response of macrophages isolated from rats fed cocoa. It has been demonstrated that these cells produced lower amounts of TNF-α, IL-6, NO, and ROS (Ramiro-Puig et al., 2007a; Castell et al., 2009). Similarly, serum concentration of MCP-1 decreased after a cocoa diet in rats (Ramos-Romero et al., 2012a).

With regard to studies in humans, it has been reported that a supplementation with cocoa products in healthy humans did not affect inflammation markers (Mathur et al., 2002); however, a cross-sectional analysis showed that the regular intake of dark chocolate by a healthy population in Southern Italy was inversely related to serum C-reactive protein concentration (di Giuseppe et al., 2008). In addition, cocoa consumption for 4 weeks decreased some adhesion molecules involved in the recruitment of inflammatory cells (Monagas et al., 2009). More recently, leukocytes from healthy volunteers showed a decrease in the activation of NF-κB and also in the serum concentrations of some adhesion molecules, such as intercellular adhesion molecule 1 and E-selectin, 6 h after receiving 40 g of cocoa powder (Vázquez-Agell et al., 2013).

### COCOA DIET AND INFLAMMATORY DISEASES

Although at present no human intervention studies applying cocoa treatment in inflammatory conditions have been reported, some studies in animal models of diseases suggest the anti-inflammatory effect of cocoa. In this context, the oral administration of a cocoa polyphenolic fraction to mice has seen to inhibit ear edema in a dose-dependent manner (Lee et al., 2006a). Moreover, rats that received cocoa for a week (4.8 g/kg/day) developed a lower paw edema induced by carrageenan and by bradykinin (Ramos-Romero et al., 2008; Castell et al., 2009).

The anti-inflammatory activity of cocoa has been extended to inflammatory bowel disease (IBD). Using IBD models, a number of flavonoids, such as quercitrin, rutin, diosmin, hesperidin, morin, and silymarin have demonstrated anti-inflammatory activity (reviewed in Comalada et al., 2013). However, a study using a cocoa diet in a dextran sodium sulfate (DSS) model demonstrated that cocoa intake did not improve clinical colitis, although it certainly contributed to reducing colonic oxidative activity and serum inflammatory mediator concentrations (Pérez-Berezo et al., 2012a). These results agree with those obtained with luteolin and with a lemon verbena infusion rich in polyphenolic compounds (Karrasch et al., 2007; Lenoir et al., 2011). More interestingly, it has recently been reported that a polyphenol-enriched cocoa extract was able to decrease acute DSS colitis in mice (Andújar et al., 2011), thus evidencing the need for a high polyphenol content in the cocoa to achieve anti-inflammatory activity in the IBD.

The effect of a cocoa diet on *in vivo* models of neuronal inflammation and systemic chronic inflammation such as adjuvant arthritis (AA) and collagen-induced arthritis (CIA) has also been reported. Rats fed a diet enriched with cocoa produced a decrease in the inflammatory response to an acute and chronic noxious stimulus of trigeminal ganglion neurons (Cady and Durham, 2010).

In AA, a cocoa-enriched diet was able to decrease the synthesis of antibodies against the pathology inducer, and to reduce the proportion of Th lymphocytes in blood and regional lymphoid tissues, but the cocoa diet produced only a tendency to modulate hind-paw swelling (Ramos-Romero et al., 2012b). It must be added that the oral administration of some flavonoids such as quercetin and hesperidin were only able to partially reduce AA swelling (Mamani-Matsuda et al., 2006; Li et al., 2010) or only slightly decreased this chronic inflammatory model (Rovenský, 2009). However, a cocoa diet was able to reduce the oxidative stress associated with AA (Ramos-Romero et al., 2012c).

Concerning the CIA model in rats, a diet enriched in cocoa beginning 2 weeks before CIA induction and given throughout the process, has been applied. Although arthritic cocoa-fed rats decreased specific autoantibody titers, the production of pro-inflammatory mediators from peritoneal macrophages, and the Th proportion in lymph nodes, they developed a similar hind-paw swelling as the reference arthritic animals (Ramos-Romero et al., 2012a). On the contrary, Miyake et al. (2008) reported that the oral administration of highly oligomeric procyanidins isolated from Jatoba (*Hymenaea courbaril*) ameliorated CIA in mice and also decreased the serum concentrations of some specific autoantibodies. Similarly, the oral consumption of an extract of green tea polyphenols reduced the incidence, the arthritis index and the autoantibody concentration of CIA in mice (Haqqi et al., 1999). Similarly, single flavonoids administered orally, such as hesperidin, resulted in preventive and therapeutic effects in mice CIA (Kawaguchi et al., 2006).

## COCOA AND LYMPHOID TISSUES

**FIGURE 1:**
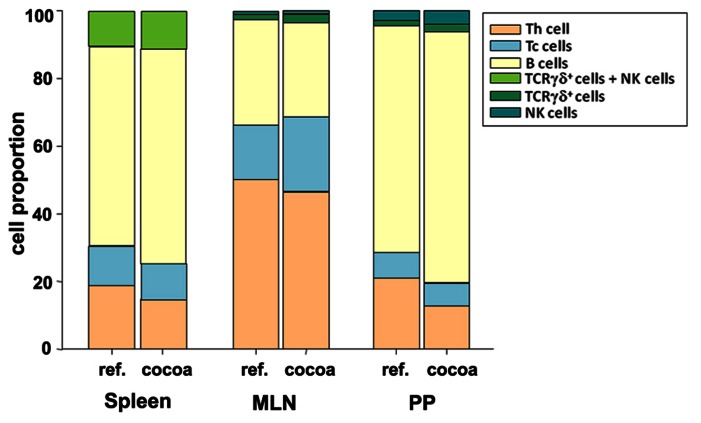
**Summary of the effects of a 10% cocoa diet in rats on lymphocyte proportion in secondary lymphoid tissues (based on Ramiro-Puig et al., 2007a, 2008).** MLN, mesenteric lymph nodes; PP, Peyer’s patches.

Primary and secondary lymphoid tissues constitute two major categories of lymphoid organs. The formation of the primary repertoire of lymphocytes takes place in the primary tissues such as thymus and bone marrow. Secondary lymphoid tissues are responsible for the coordination of immune responses by spatially organizing the interaction of immune effector cells (Drayton et al., 2006). By means of preclinical studies in rats, it has been evidenced that a cocoa diet can induce changes in the cell composition of both primary and secondary lymphoid organs. In particular, a cocoa diet has an influence on the proportion of B lymphocytes and T cell subsets, i.e., T cell receptor (TCR) αβ^+^ cells, TCRγδ^+^ cells, T helper (Th), cells and T cytotoxic (Tc) cells (**Figure 1**).

A cocoa diet influences antioxidant status and the cell composition of rat thymus. A diet containing 10% cocoa in rats increased the thymic content of catalase and superoxide dismutase and promoted the progression of immature thymocytes (double negative TCRαβ^low^ and double positive TCRαβ^low^ cells) toward more mature T cell stages (CD4^+^CD8^-^ TCRαβ^high^ cells; Ramiro-Puig et al., 2007b). Similarly, a diet with 10% cocoa was able to influence a secondary lymphoid tissue such as the rat spleen and lymph nodes (**Figure 1**). Young rats fed cocoa decreased the spleen percentage of Th cells while increasing that of B cells (Ramiro-Puig et al., 2007a). Additionally, adult Louvain rats fed 10% cocoa for 6 weeks reduced the proportion of TCRαβ^+^ cells in inguinal lymph nodes (Ramos-Romero et al., 2012a). Likewise, the percentage of Th cells was reduced in mesenteric lymph nodes (MLNs) at the expense of Tc cells that increased in young Wistar rats fed 10% cocoa for 3 weeks, but not 4% cocoa (Ramiro-Puig et al., 2008; **Figure 1**). A high-cocoa diet also affects the lymphocyte composition of intestinal Peyer’s patches (PPs). In particular, cocoa intake reduced the TCRαβ^+^ cell percentage, mainly due to a decrease in the Th cell proportion, and increased B cell and TCRγδ^+^ cell percentages (Ramiro-Puig et al., 2008; **Figure 1**). The increase in TCRγδ^+^ cell percentages in PPs and MLNs induced by cocoa is similar to the effects of apple polyphenol intake in healthy mice (Akiyama et al., 2005) and could be especially important during childhood, when the immune system is maturing (Pérez-Cano et al., 2005), or in the prevention of food allergies.

## INFLUENCE OF COCOA ON ADAPTIVE IMMUNE RESPONSE

The adaptive immune response is an intricate reaction comprising a number of intracellular and intercellular events from the antigen entry until the development of effector mechanisms. Dendritic cells (DC), acting as antigen-presenting cells, take up, process and present antigen to TCR-specific Th lymphocytes. The interaction between DC and Th cells involves a lot of co-stimulatory molecules thus forming the immune synapses (Dustin and Groves, 2012). Next, Th lymphocytes proliferate and differentiate, becoming effector cells such as Th1, Th2, Th17, or regulatory T cells that produce cytokines. Some of these cytokines involve the activation of other antigen-specific cells such as Tc cells or B cells. Activated B cells differentiate into plasma cells, which synthesize antibodies that specifically bind the antigen that has triggered the adaptive immune response.

The first event in adaptive response refers to Th cell activation. Specific recognition of antigenic peptide by TCR together with co-stimulatory molecules causes production of IL-2 (Malek, 2008). IL-2 binds to a receptor consisting of the subunits α, β, and γ (CD25, CD122, and CD132, respectively) to produce cell proliferation (Boyman and Sprent, 2012). CD25 is expressed after Th cell activation. The binding of IL-2 to its receptor leads to the stimulation of complex transduction signals involving mitogen-activated protein kinase (MAPK), Janus kinase/signal transducer and activator of transcription (JAK/STAT), and phosphatidylinositide 3-kinase (PI3K)/Akt pathways that eventually mediates multiple biological processes including T cell and B cell growth and differentiation (Malek, 2008; Boyman and Sprent, 2012).

### COCOA AND Th CELL ACTIVATION

IL-2 and CD25 are markers of early lymphocyte T activation. Some *in vitro* studies have reported the effect of isolated cocoa flavonoids and cocoa extracts in the synthesis of IL-2. Sanbongi et al. (1997) found that cocoa liquor polyphenols inhibited both IL-2 gene expression and IL-2 secretion in human blood T cells. Likewise, Mao et al. (1999, 2000) showed that a crude cocoa extract and pentamer, hexamer and heptamer procyanidins from cocoa also reduced IL-2 transcription in phytohemagglutinin (PHA)-stimulated human PBMC. Similarly, in a lymphoid cell line activated with phorbol 12-myristate 13-acetate (PMA) and IL-1 and cultured in the presence of epicatechin or a cocoa extract, it has been established that cocoa flavonoids were able to decrease the expression of surface CD25 and to diminish IL-2 secretion (Ramiro et al., 2005b). The ability of the cocoa extract to decrease CD25 expression was higher than that of epicatechin alone, which may be due to the effect of other cocoa flavanols (Ramiro et al., 2005b). Overall, these *in vitro* studies agree that cocoa flavonoids can decrease IL-2 production in Th cells. These results are also in line with those described with other flavonoids, such as genistein (Atluru et al., 1991) and Pycnogenol0^®^ (Cho et al., 2001). How cocoa flavonoids modulate IL-2 gene is not known but it has been demonstrated that epicatechin and dimeric procyanidins decrease NF-κB activation on PMA-activated Jurkat cells, a lymphoid cell line (Mackenzie et al., 2004). The inhibition of NF-κB might mediate the downregulation of both IL-2 and CD25 in a similar way to that of the decrease in pro-inflammatory mediators.

It is interesting to note the effect of cocoa procyanidins on the plasma membrane of Jurkat T cells. After the adsorption of flavonoids, the plasma membrane became more fluid, and procyanidins prevented the leakage of small molecules from vesicles (Verstraeten et al., 2004). These effects could also influence the establishment of immune synapses thus attenuating the interaction between the Th cell and the antigen-presenting cell.

In spite of the *in vitro* results, *in vivo* studies do not confirm the downregulation of IL-2 by cocoa flavonoids. Some studies carried out in rats showed the effect of a diet containing cocoa on the functionality of immune cells isolated from spleen or lymph nodes. Splenocytes from rats fed cocoa (a diet with either 4 or 10% defatted cocoa) did not decrease IL-2 production or CD25 surface expression after stimulation with PMA plus ionomycin (Ramiro-Puig et al., 2007a; Pérez-Berezo et al., 2009). Likewise, these cells showed a similar or even higher proliferative response (Ramiro-Puig et al., 2007a). In the same way, lymphocytes from cocoa-fed rat MLN produced higher or equal amounts of IL-2 (Ramiro-Puig et al., 2008; Pérez-Berezo et al., 2009; Ramos-Romero et al., 2012b).

### COCOA AND EFFECTOR T CELLS

After naïve Th cell activation and proliferation, effector Th lymphocytes appear. Depending on the cytokines released to the medium, which are eventually related to the antigen that trigger the immune response, activated Th1 cells, Th2 cells, Th17 cells, or regulatory T cells result (Nakayama and Yamashita, 2008; Amsen et al., 2009; Korn et al., 2009). Th1 cells direct cell-mediated immunity against intracellular pathogens by means of the synthesis and release of interferon (IFN)-γ, TNF-α, and TNF-β, among others. These cytokines promote phagocytosis and cytotoxicity recruiting macrophages, natural killer (NK) cells, Tc cells, and also the enhancement of complement-activating antibodies synthesis. Th1 activity is usually associated with inflammation (Nakayama and Yamashita, 2008). Th2 cells are designed to fight against extracellular pathogens, activating mast cells and eosinophils, and the production of antibodies which are not able to activate the complement system. Th2 cells are involved in the humoral immunity and allergic reactions (Nakayama and Yamashita, 2008). The Th2 subset produces cytokines such as IL-4 and IL-5 that help B cells to proliferate and differentiate, and IL-10 with anti-inflammatory properties. IL-4 is mainly produced by activated Th2 cells and plays an important role in regulating Th1/Th2 balance (Nakayama and Yamashita, 2008). Recently, the effectors Th cell family expanded with the discovery of Th17 cells. These cells produce IL-17 and exhibit effector functions distinct from Th1 and Th2 cells. The primary function of Th17 cells appears to be the clearance of pathogens that are not adequately handled by Th1 or Th2 cells and they are potent inducers of tissue inflammation (Korn et al., 2009).

The effect of cocoa diets in rats on the cytokine production by Th1 and Th2 cells has been reported. The secretion of IFN-γ, the main cytokine related to Th1 activity, has been quantified in cells isolated from the spleen and lymph nodes of rats fed a cocoa diet. No changes in the secretion of this cytokine were observed in splenocytes (Ramiro-Puig et al., 2007a; Ramos-Romero et al., 2012b), although others found increased values (Pérez-Berezo et al., 2009), and *in vitro* studies demonstrated a suppression of IFN-γ production by PHA-stimulated PBMC (Jenny et al., 2009).

More interestingly, a cocoa diet in rats produced a lower IL-4 secretion in isolated splenocytes (Ramiro-Puig et al., 2007a; Pérez-Berezo et al., 2009) and MLN cells (Ramiro-Puig et al., 2008). However, IL-10 secretion was not modified in rats fed a cocoa diet (Ramiro-Puig et al., 2007a, 2008). The results obtained in these *in vivo* experiments did not exactly fit with those obtained in *in vitro* studies with cocoa flavonoids. Thus, an increase in IL-4 secretion after epicatechin addition in a lymphoid cell line and PBMC has been reported (Mao et al., 2000; Ramiro et al., 2005b), whereas hexamer to octamer cocoa procyanidins presented an inhibitory effect on this cytokine (Mao et al., 2000).

### COCOA AND HUMORAL IMMUNE RESPONSE

As stated before, an increase in the percentage of B cells in spleen was observed in rats fed cocoa (**Figure 1**). However, the antibody response of these cells has been found to be attenuated. Thus, the ability to produce IgG, IgM, and IgA by splenocytes from rats fed cocoa was depressed (Ramiro-Puig et al., 2007a). This effect was also reflected in serum immunoglobulin concentrations. Three-week-old rats fed with 10% cocoa for 3 weeks, but not those fed 4% cocoa, had lower serum IgG, IgM, and IgA concentrations (Ramiro-Puig et al., 2007a; Pérez-Berezo et al., 2012b). However, when the cocoa diet began later and the dose was lower, the effect was not so patent (Pérez-Berezo et al., 2011).

The influence of a 4 and 10% cocoa diet on the antibody synthesis in immunized rats has been reported. When animals were fed cocoa before and during an immunization process, the synthesis of specific antibodies and the number of IgG-secreting cells decreased, although the proliferation rate of lymph node and spleen cells was maintained (Pérez-Berezo et al., 2009). The analysis of antibodies demonstrated that the impact on humoral response did not affect all antibody isotypes equally. The most attenuated isotypes were specific IgM, IgG1, IgG2a, and IgG2c whereas anti-OVA IgG2b concentrations held steady or increased with the 10% cocoa diet. IgG isotypes can be associated with Th1 or Th2 immunity. In the rat, IgG1 and IgG2a are related to the Th2 response, while IgG2b depends on the Th1 response (Binder et al., 1995; Gracie and Bradley, 1996; Saoudi et al., 1999; Bridle et al., 2007). These results agree with others that evaluated certain food polyphenols, such as those from apple or soybean (Akiyama et al., 2005; Kogiso et al., 2006). From all these results, it has been suggested that a cocoa diet mainly downregulates the Th2 immune response, whereas it maintains Th1 immunity. This hypothesis was supported by a lower IL-4 secretion from splenocytes and a higher production of IFN-γ from lymph node cells (Pérez-Berezo et al., 2009).

Because of a cocoa diet seems to attenuate antibody synthesis, it has been tested in experimental disease models in which antibodies play a pathogenic role, such as autoimmune diseases and allergic processes.

Rheumatoid arthritis (RA) is a systemic autoimmune disease in which chronic inflammation of synovial joints results in joint destruction, pain, disability, and a reduced life expectancy (Wegner et al., 2010). The pathology of the RA is mediated by specific autoantibodies, mainly against citrullinated proteins such as collagen type II (Wegner et al., 2010). In consequence, CIA in rats or mice is the gold standard *in vivo* model for RA studies (Asquith et al., 2009). In such rat experimental model, the influence of a cocoa diet on joint inflammation and autoantibody titers has been reported (Ramos-Romero et al., 2012a). Louvain rats fed cocoa from 2 weeks before arthritis induction, and during the disease period studied (4 weeks), reduced the synthesis of specific antibodies against type-II collagen, but this effect was not enough to mitigate the hind-paw swelling in arthritic animals during the study period (Ramos-Romero et al., 2012a).

Allergic reactions are mainly caused by IgE-mediated hypersensitivity. In allergic patients, the immune system reacts to innocuous substances by producing IgE. These antibodies bind to mast cells and, after allergen reaction, produce degranulation of mast cell mediators with a subsequent generation of allergic manifestations (Amim, 2012). The effect of cocoa in an allergy model has been preclinically studied. A diet containing 10% cocoa prevented the synthesis of antibodies involved in allergic reaction in young rats, in particular, rats fed a cocoa diet showed lower titers of specific IgG1, IgG2a and a decrease of specific IgE of about 60–70% (Abril-Gil et al., 2012). The effect of a cocoa diet on allergic manifestations has not yet been established. However, it is noteworthy that the modulation of specific IgE was also observed in allergy models after treatment with flavonoids, and in these studies the effect on IgE synthesis was associated with lower allergy signs. This is the case in treatment with luteolin (Das et al., 2003), baicalin (Won Jung et al., 2012), biochanin A (Ko et al., 2011), quercetin (Cruz et al., 2012), myricetin (Medeiros et al., 2008), and hesperidin (Rogerio et al., 2007; Joskova et al., 2011). In addition, it can be added that clinical trials applying a treatment with Pycnogenol^®^, an extract of *Pinus maritime* containing procyanidins, demonstrated the efficacy of such intervention in reducing some signs of allergic asthma (Hosseini et al., 2001; Lau et al., 2004; Belcaro et al., 2011). From all these studies it can be concluded that foods enriched in flavonoids, such as cocoa, open a new perspective in their use as a nutraceutical in allergic diseases.

## EFFECTS OF COCOA ON INTESTINAL IMMUNITY

The digestive system is the first compartment reached by dietary compounds. Bacteria, epithelial cells, and immune cells in the intestine are the first ones to be affected by diet. Every day, the gut-associated lymphoid tissue (GALT), which constitutes the most extensive and complex part of the immune system in the body, receives a huge antigenic load and has to distinguish between invasive pathogens and innocuous antigens from food and commensal bacteria. Briefly, the intestinal immune response is initiated in the M cells from PPs which uptake luminal antigens and transport them toward DC, which interact with interfollicular T lymphocytes or migrate toward MLN (Cerutti and Rescigno, 2008). This process induces differentiation and maturation of B cells, which become IgA^+^ cells and later IgA-secreting cells (Kunisawa and Kiyono, 2005). The main resulting product of the GALT is the secretory-IgA (S-IgA; Mora and von Andrian, 2008; Brandtzaeg, 2010). This immunoglobulin constitutes the first line of non-inflammatory immune protection at mucosal surfaces by neutralizing microbial pathogens and exotoxins and by interacting with innocuous dietary antigens and commensal microbes (Corthésy, 2007; MacPherson et al., 2008).

**FIGURE 2:**
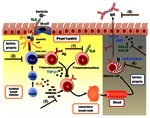
**Summary of the effects of a 10% cocoa diet in rat’s gut-associated lymphoid tissue (Ramiro-Puig et al., 2008; Fagarasan et al., 2010; Pérez-Berezo et al., 2011; Pérez-Berezo et al., 2012b).** Generation of S-IgA can be developed by a T cell-dependent process initiated in Peyer’s patches or by a T cell-independent mechanism in isolated lymphoid follicles and lamina propria. Both mechanisms lead to the induction of IgA^+^ B cells which migrate to blood and come back to the gut lamina propria where they differentiate into plasma cells and produce S-IgA. The mechanisms showing how cocoa modulates immune response are shown in the figure with numbers in brackets, and as follows: cocoa induces a differential pattern of Toll-like receptor (TLR) gene expression (1) which may interfere with both dependent and independent pathways. Moreover, Th2 development and conventional B–T cell interactions through major histocompatibility complex (MHC)-TCR and CD40–CD40L are also modulated by cocoa (2). The preferential generation of IgA^+^ B cells is caused by the abundant production of activated TGF-β1. Cocoa compounds also influence the migration of IgA^+^ B cells into the gut lamina propria, by modifying the expression of some chemokines (CCL25, CCL28) (5) or their receptors (CCR9) (4) whose expression depends on retinoic acid (3). As a result, the amount of S-IgA in the intestinal lumen in cocoa-fed animals is markedly reduced (6).

Few studies addressing the dietary effects of cocoa on GALT function in healthy animals or humans have been reported to date. Dietary intervention with cocoa did not morphologically affect the intestinal structure (Ramiro-Puig et al., 2008), but is capable of modifying some important aspects of the GALT composition and functionality in rats as next detailed (Ramiro-Puig et al., 2008; Pérez-Berezo et al., 2011, 2012b; **Figure 2**).

### COCOA DIET AND MESENTERIC LYMPH NODE LYMPHOCYTE ACTIVATION

Rat interventional nutrition with a cocoa diet modulates MLN lymphocyte activation in certain conditions. Isolated MLN cells from young rats fed 10% cocoa for 3 weeks strongly enhanced IL-2 secretion; nevertheless, the proliferation rate did not increase after 48 h of cell culture (Ramiro-Puig et al., 2008). On the other hand, isolated MLN lymphocytes from rats fed a long-term cocoa diet (9 weeks) did not change IL-2 production after *in vitro* mitogen activation nor their proliferative ability after *in vitro*-specific activation (Pérez-Berezo et al., 2009).

To ascertain whether cocoa modified Th effector cell functionality in the GALT, IFNγ, IL-4, and IL-10 cytokine production was studied in stimulated MLN cells isolated from animals fed a cocoa diet (Ramiro-Puig et al., 2008). The nutritional 10% cocoa intervention for 3 weeks resulted in a lower IL-4 secretion, IL-10 secretion tended to decrease whereas that of IFN-γ tended to increase. A 4% cocoa intake for 3 weeks did not produce any significant modification (Ramiro-Puig et al., 2008). These results suggest that high-cocoa diets, similarly to the results found in the systemic compartment, downregulate Th2 responses, and therefore, may downregulate B cell differentiation and immunoglobulin production even at mucosal sites.

### COCOA AND SECRETORY-IgA

Secretory-IgA plays a key role in the maintenance of gut homeostasis and oral tolerance and its function and production are tightly regulated (Cerutti and Rescigno, 2008). The relationship between a cocoa diet and S-IgA has been demonstrated in different experimental designs using rats, where the effect of varied proportions of cocoa diets (2, 4, 5, and 10%), different age at the beginning of the dietary nutritional (3 or 6 weeks of age) and length of diet (3, 6, or 9 weeks) have been analyzed. As demonstrated next, cocoa reduces S-IgA protein and gene expression which conduces a different pattern of IgA-coating bacteria. The effect of cocoa on S-IgA might be due to the influence of cocoa on genes related to Th maturation, Th–B cell interactions, and IgA^+^ B cell gut-homing, among others (Pérez-Berezo et al., 2011, 2012b).

First data showing a downmodulatory effect of cocoa on S-IgA were established in fecal samples after 2 weeks of both 4 and 10% cocoa intake in young rats. However, this effect only remained for 3 weeks in the 10% cocoa diet. The decrease in fecal IgA correlated with a lower concentration of S-IgA and S-IgM in gut washes (Ramiro-Puig et al., 2008). These results were confirmed in a study that extended the dietary intervention with a 10% cocoa diet up to 6 weeks (Pérez-Berezo et al., 2012b). If dietary intervention began later in rat age (6-week-old animals), the attenuating effect of cocoa remained for 5 and 10% cocoa diet, showing a rapid effect even 1 week after the diet start (Pérez-Berezo et al., 2011). However, a lower cocoa proportion (2%) tended to reduce fecal IgA levels after only 3 weeks of diet. Therefore, cocoa diets, especially those with a higher cocoa proportion, decreased S-IgA concentration in the intestinal lumen of rats (Ramiro-Puig et al., 2008). This effect was associated with a lower number of PP cells with a high capacity to secrete IgA (Ramiro-Puig et al., 2008), and with a downregulation of IgA gene expression in PP cells and in the wall of the small intestine and colon (Pérez-Berezo et al., 2011, 2012b; Massot-Cladera et al., 2012).

Some commensal intestinal bacteria in humans and rodents coat S-IgA by an apparent non-random immunological phenomenon (Tsuruta et al., 2010). In fecal samples from rats collected before and after a cocoa diet, the IgA-coating bacteria were enumerated. After 6 weeks, reference animals showed an increase in the percentage of IgA-coating bacteria that was avoided with the 10% cocoa diet (Massot-Cladera et al., 2012).

In order to look further into the downregulation of S-IgA through a cocoa diet in rats, the gene expression of several molecules involved in intestinal immune response was established using different cocoa proportions (2, 5, and 10%), supplementation periods (3 or 6 weeks) and initial age of rats (3 or 6 weeks; Pérez-Berezo et al., 2011, 2012b). A pathway for B cells to become IgA-secretory cells is a T cell-dependent process located in either PPs or MLNs, inductive sites of the intestinal immune system (Kunisawa and Kiyono, 2005; Mora and von Andrian, 2008). The maturation of mucosal Th cells depends on IL-6, among others; the interaction between activated Th cells and B cells requires the interaction of CD40 ligand with CD40 (Islam et al., 1991; Cerutti, 2008), and the differentiation of B cells into IgA^+^ B cells involves transforming growth factor-β1 (TGF-β1), IL-5, IL-6, IL-10, and IL-21 (Schoenbeck et al., 1989; Brière et al., 1994; Ramsay et al., 1994; Dullaers et al., 2009). The study of a 10% cocoa diet for 3 or 6 weeks in rats on the mechanisms of S-IgA secretion revealed that the cocoa diet did not modify TGF-β1 gene expression in PPs, MLNs, or the small intestine; however, IL-6 gene expression was reduced ~95% in MLNs after 6 weeks of a 10% cocoa diet (Pérez-Berezo et al., 2012b) but not earlier (Pérez-Berezo et al., 2011). Likewise, cocoa intake did not modify CD40 gene expression either in PPs or in MLNs (Pérez-Berezo et al., 2012b), which is in accordance with previous studies that have shown that a cocoa diet increased the proportion of B cells in PPs (Ramiro-Puig et al., 2008); however, a 10% cocoa diet (but not lower proportions) for 6 weeks (but not in a shorter period), reduced CD40 gene expression in the small intestine (Pérez-Berezo et al., 2011, 2012b).

When IgA^+^ B cells become activated they leave PPs, go to the bloodstream and come back to the intestine or other mucosa (MacPherson et al., 2008; Brandtzaeg, 2010). The gut-homing system requires the integrin α4β7 and some chemokine receptors on activated gut lymphocytes (Mora and von Andrian, 2008). Chemokines produced by epithelial cells such as CCL25 and CCL28 interact with the chemokine receptors CCR9 and CCR10 respectively, to recruit IgA^+^ B cells (Hieshima et al., 2004). The CCR9 expression on IgA^+^ B cells is induced by retinoic acid (Mora et al., 2006) through its ligation to nuclear retinoic acid receptors (RAR; Ross et al., 2009). Diets containing 2, 5, or 10% cocoa for 3 weeks in 6-week-old animals did not affect the gene expression of CCR9, CCL25, RARα, or RARβ but increased the CCL28 gene expression in the small intestine wall (Pérez-Berezo et al., 2011). The increase in the CCL28 gene expression could reflect a “rescue mechanism” to strongly attract the IgA^+^ B cells to the gut, in an attempt to compensate the S-IgA downregulation. When the cocoa diet began earlier and lasted longer (3-week-old animals fed 10% cocoa for 6 weeks), the gene expression of gut-homing molecules such as RAR, CCR9, and CCL28, but not CCL25, was downregulated in the small intestine (Pérez-Berezo et al., 2012b). Overall, these results demonstrate the longer the cocoa intake the greater the sensitivity of gut-homing mechanisms in the intestine.

Finally, when IgA^+^ B cells reach the intestine, they differentiate into IgA-secreting cells mainly releasing dimers of IgA. This immunoglobulin is actively secreted to the apical surface of epithelial cells by a polymeric immunoglobulin receptor (pIgR) expressed on the basolateral surface of epithelial cells (Cerutti and Rescigno, 2008). The gene expression of pIgR was not modified by any cocoa diet given for 3 or 6 weeks (Pérez-Berezo et al., 2011, 2012b).

In summary, a high-cocoa diet induces a lower number of IgA^+^ B cells reaching the intestinal lamina propria by downregulating either the expression of chemokine or that of their receptors (**Figure 2**). However, in the gut lamina propria some other mechanisms remain working efficiently.

### COCOA AND BACTERIA RECOGNITION

The GALT maintains mucosal homeostasis by inducing a state of non-responsiveness to innocuous antigens, such as commensal bacteria, or by responding actively to counteract pathogens (Fagarasan et al., 2010). In this regard, toll-like receptors (TLRs), through the recognition of conserved molecular motifs on microorganisms, are important molecules involved in the cross-talk between microorganisms and gut epithelial and immune cells (Abreu, 2010). It has been reported that the generation of IgA^+^ B cells can be independent of Th cells and involve TLR non-specific recognition (Fagarasan et al., 2010). In this context, cocoa diets in rats have shown differential TLR expression patterns for TLR2, TLR4, TLR7, and TLR9 in PPs, MLNs, the small intestine and colon (Pérez-Berezo et al., 2011, 2012b; Massot-Cladera et al., 2012).

A high (10%) and continuous cocoa diet produced an upregulation of TLR4 and TLR9 and a downregulation of TLR2 and TLR7 in PPs and MLNs (inductor sites of intestinal immune response; Pérez-Berezo et al., 2011, 2012b). Conversely, in the small intestine and the colon, cocoa-fed animals showed lower TLR4 and TLR9 and higher TLR2 and TLR7 gene expression (Massot-Cladera et al., 2012; Pérez-Berezo et al., 2012b). TLR4 expression is positively correlated to the number of IgA-secreting cells in the lamina propria and their recruitment through CCL28 (Shang et al., 2008; Abreu, 2010). In consequence, the downregulation of TLR4 through a cocoa diet in the intestine (as effector site) could be associated with the decrease in S-IgA in feces.

Toll-like receptors are expressed preferentially in tissues that are in constant contact with microorganisms (Cario, 2005; Abreu, 2010). Therefore, changes in the TLR expression could reflect changes in the intestinal microbiota and/or its relation to intestinal immune cells (Shibolet and Podolsky, 2007).

## INFLUENCE OF COCOA ON GUT MICROBIOTA

**FIGURE 3:**
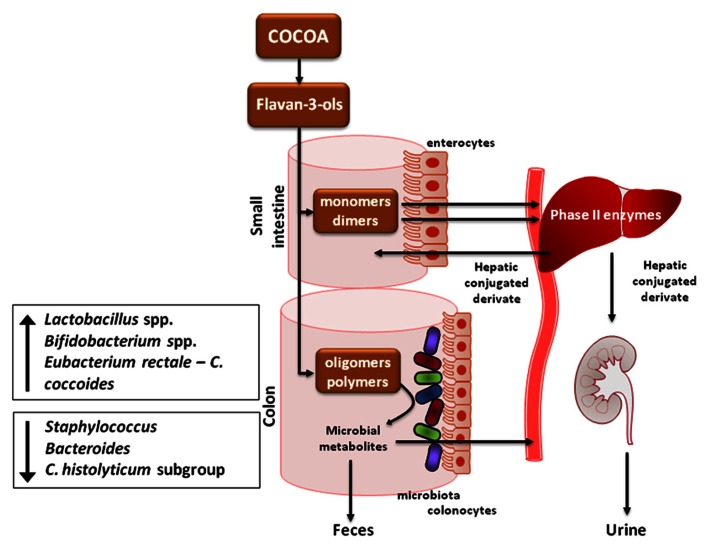
**Metabolic route of consumed cocoa flavonoids, and effect on intestinal microbiota (based on Tzounis et al., 2008, 2011; Monagas et al., 2010; Massot-Cladera et al., 2012).** Flavanol monomers and dimers are absorbed in the small intestine, while procyanidins reach the colon and are metabolized by the intestinal microbiota into various phenolic acids that are later absorbed. Absorbed compounds are metabolized in the liver and subsequently eliminated in urine. A portion of microbial metabolites is eliminated in the feces. Cocoa flavonoids decrease the proportion of *Bacteroides*, *Staphylococcus* genus, and *C. histolyticum* subgroup whereas they enhance the growth of *Lactobacillus* spp. and *Bifidobacterium* spp., and *Eubacterium rectale–C. coccoides*.

The above data demonstrate that cocoa-enriched diets in rats influence the intestinal immune system either through a direct effect on intestinal immune cells and/or an indirect effect mediated by changes in microbiota which would influence the cross-talk with the host (i.e., through TLR). Therefore, it remained to be seen whether cocoa diets modulated microbiota composition and, consequently, the intestinal immune system. This is possible due that cocoa flavonoids reaching the colon can interact with intestinal microbiota through a bidirectional relationship. Thus, bacteria can be involved in the polyphenol metabolism, and flavonoids can influence microbiota growth and composition (Hayek, 2013). In this context, changes in intestinal microbiota composition may influence the immune system as well as the compounds originated by the bacterial metabolism (**Figure 3**).

### COCOA FLAVONOIDS METABOLISM

Cocoa flavonoids have a particular bacterial metabolism due to the high degree of polymerization of its flavanols. After cocoa intake, monomers (i.e., catechin and epicatechin) are rapidly absorbed in the small intestine, while the largest proportion of dietary polyphenols (90–95%) in the form of oligomers and polymers (i.e., cocoa procyanidins) pass intact through the gastrointestinal tract, reaching the colon (Monagas et al., 2010). This fact allows them to be metabolized by the intestinal microbiota (**Figure 3**). Colonic bacteria is composed of more than 500 species and a bacterial load of approximately 10^11^ to 10^12^ bacteria/g of colonic contents (O’Hara and Shanahan, 2006). It is known that microbiota has the ability to metabolize polyphenols to simpler metabolites and this conversion is often essential for absorption and modulates the biological activities of these compounds which are more beneficial than the original forms found in food (Clifford, 2004; Tzounis et al., 2008; Selma et al., 2009; Monagas et al., 2010; Neilson and Ferruzzi, 2011).

Cocoa polyphenols are extensively degraded in the colon by a broad range of reactions able to generate various phenolic acids, mainly including phenylpropionic, phenylacetic, and benzoic acid derivates (Déprez et al., 2000; Kohri et al., 2003; Aura, 2008; Tzounis et al., 2008; Fogliano et al., 2011). Later, colon bacterial metabolites are absorbed into the bloodstream, providing another source of potentially bioactive compounds (Rios et al., 2003). Once absorbed, the microbial metabolites from flavanols are mainly metabolized in the liver by phase-II enzymes as hepatic conjugated derivatives that are subsequently eliminated in urine (Neilson and Ferruzzi, 2011; **Figure 3**). In particular, the presence of 5-(3′,4′,5′-trihydroxyphenyl)-γ-valerolactone and 5-(3′,4′-dihydroxyphenyl)-γ-valerolactone in urine is considered to be a potential biomarker of flavan-3-ols consumption in humans after cocoa products intake (Urpi-Sarda et al., 2009). At the same time, a portion of microbial metabolites (non-conjugated microbial metabolites) is eliminated in the feces. The excretion of microbial metabolites varies markedly between subjects and, for some individuals, it may also vary with the substrate (Monagas et al., 2010).

Regarding the intestinal bacteria with the ability to catabolize flavanols, a limited number of bacterial species have been identified as being involved in the polyphenols catabolism. Interestingly, the majority of the bacteria characterized belong to the Clostridia group, which is a large component of the gut microbiota (Wang et al., 2001; Tzounis et al., 2008).

### COCOA INFLUENCE ON GUT MICROBIOTA COMPOSITION

It is known that unabsorbed dietary phenolics and their metabolites can exert significant effects on the intestinal environment by modulation of the microbiota (Lee et al., 2006b). Although there is limited information concerning the ability of (+)-catechin and (-)-epicatechin, the main monomers present in cocoa, to promote or inhibit the growth of selected intestinal bacteria, there are some *in vitro*, preclinical and clinical studies regarding this subject (Tzounis et al., 2008, 2011; Massot-Cladera et al., 2012).

*In vitro* studies have shown the antimicrobial properties of some polyphenols (Puupponen-Pimiä et al., 2005; Lee et al., 2006b). To date, Tzounis et al. (2008) showed that (+)-catechin induced an inhibitory effect in the growth of the *Clostridium histolyticum* group using the batch culture approach, at the same time that both (+)-catechin and (-)-epicatechin enhanced the growth rate of the beneficial bacteria group, *Eubacterium rectale–C. coccoides*. Furthermore, there were increases in both *Lactobacillus* spp. and *Bifidobacterium* spp. genus following (+)-catechin exposure, as well as a small but significant increase in the growth of *E. coli* after (+)-catechin incubation (Tzounis et al., 2008; **Figure 3**).

The effects of cocoa polyphenols observed in animal models are partially in line with the above results. Young rats receiving a 10% cocoa intake for 6 weeks showed a significant decrease in the proportion of *Bacteroides*, *Staphylococcus* genus, and *C. histolyticum* subgroup (Massot-Cladera et al., 2012). The effect of this defatted cocoa powder on microbiota was also observed in rats fed for 4 weeks with diets based on cocoa polyphenols-enriched powders (Massot-Cladera et al., 2013).

With regard to human studies, evidence of the effects of cocoa or cocoa products intake on microbiota composition is scarce. A human intervention study evaluated the high-cocoa flavanol consumption effect on microbiota composition from healthy volunteers (Tzounis et al., 2011). The results showed that a 4 weeks daily ingestion of a high-cocoa flavanol beverage containing 494 mg flavanols increased the growth of *Lactobacillus* spp., and *Bifidobacterium* spp. in comparison with a control low-cocoa flavanol drink that contained only 29 mg flavanols (Tzounis et al., 2011). Although these results were not found in the preclinical interventions, it prompted the redefinition of cocoa polyphenols as prebiotics. It is important to mention that divergence between the animal and human data could be ascribed to several factors, such as the cocoa composition (fiber and flavonoids pattern), dose, and differential composition and distribution ecosystem (rats vs human gut). Overall, all these findings strengthen the evidence that cocoa polyphenols can have significant effects on the growth of select gut microbiota (**Figure 3**).

## CONCLUDING REMARKS

A cocoa diet has been shown to influence the immune system: in the innate inflammatory response as well as in the adaptive immunity, and in both systemic and intestinal compartments.

From the results concerning the effects of cocoa on inflammatory reaction, it could be concluded that although cocoa demonstrates clear anti-inflammatory properties *in vitro*, when tested *in vivo* results are more controversial. To date, it can be concluded that if inflammation is mild and cocoa has a high polyphenol content, it could help in the resolution of inflammatory response, and, in any case, due to its antioxidant properties, cocoa can be a complementary anti-inflammatory therapy.

Concerning lymphocytes and adaptive immune response, cocoa diet in young rats influences lymphoid tissue composition mainly by decreasing the proportion of Th cells by unknown mechanisms. In addition, the influence of cocoa on the first phases of T lymphocyte activation is not clear. *In vitro* studies show the downregulatory effect of cocoa on IL-2 production, but is not confirmed *in vivo*. When studying effector Th cells, it seems clear that in rat, a diet containing 10% cocoa produces a downregulation of Th2 response. In addition, it is worth noting the effect of this diet in attenuating the synthesis of antibodies. The downregulatory effects of cocoa could then be applied to counteract immune-mediated diseases in which antibodies play a pathogenic role. In autoimmune inflammatory diseases, a cocoa diet does not potently reduce inflammation but counteracts concomitant oxidative stress. More interestingly, a cocoa diet in rats reduces IgE synthesis which could be useful in treating allergic diseases.

In addition to these effects, a cocoa diet also influences the functionality of gut-associated lymphoid tissue. Here, similarly to the results found in the systemic compartment, a cocoa diet in rats downregulates Th2 responses and also the intestinal immunoglobulin production. It has been demonstrated that a cocoa diet influences some intercellular reactions and the gut-homing process of activated cells, resulting, therefore, in an attenuation in the synthesis of S-IgA into the intestinal lumen. Moreover, a cocoa diet is able to modify intestinal microbiota and also the cross-talk between these bacteria and body cells.

All these results allow us to suggest that a cocoa diet could be beneficial in certain immune processes. Further research may elucidate the cocoa compounds involved in such an effect and also the possible medical approaches to these repercussions.

## Conflict of Interest Statement

The authors declare that the research was conducted in the absence of any commercial or financial relationships that could be construed as a potential conflict of interest.
